# Alterations of the Murine Gut Microbiome with Age and Allergic Airway Disease

**DOI:** 10.1155/2015/892568

**Published:** 2015-05-18

**Authors:** Marius Vital, Jack R. Harkema, Mike Rizzo, James Tiedje, Christina Brandenberger

**Affiliations:** ^1^Center for Microbial Ecology, Michigan State University, East Lansing, MI 48824, USA; ^2^Department of Medical Microbiology, Helmholtz Centre for Infection Research (HZI), 38124 Braunschweig, Germany; ^3^Pathobiology and Diagnostic Investigation, Michigan State University, East Lansing, MI 48824, USA; ^4^Institute of Functional and Applied Anatomy, Hannover Medical School, 30625 Hannover, Germany

## Abstract

The gut microbiota plays an important role in the development of asthma. With advanced age the microbiome and the immune system are changing and, currently, little is known about how these two factors contribute to the development of allergic asthma in the elderly. In this study we investigated the associations between the intestinal microbiome and allergic airway disease in young and old mice that were sensitized and challenged with house dust mite (HDM). After challenge, the animals were sacrificed, blood serum was collected for cytokine analysis, and the lungs were processed for histopathology. Fecal pellets were excised from the colon and subjected to 16S rRNA analysis. The microbial community structure changed with age and allergy development, where alterations in fecal communities from young to old mice resembled those after HDM challenge. Allergic mice had induced serum levels of IL-17A and old mice developed a greater allergic airway response compared to young mice. This study demonstrates that the intestinal bacterial community structure differs with age, possibly contributing to the exaggerated pulmonary inflammatory response in old mice. Furthermore, our results show that the composition of the gut microbiota changes with pulmonary allergy, indicating bidirectional gut-lung communications.

## 1. Introduction

The gut microbiota plays a critical role in maintaining intestinal homeostasis in healthy individuals. It sustains the colonic energy balance and directly interacts with intestinal immunity (reviewed in [1]) via various immunomodulatory metabolites such as short chain fatty acids (SCFA) that support immunological tolerance and maintain the inflammatory equilibrium [2, 3]. However, immune modulation by the gut microbiome is not restricted to the intestinal environment, but also affects peripheral organs including the lung [4]. Changes in the gut microbiota and resulting alterations in immune function have been associated with various pulmonary diseases. For instance, disturbance of the commensal bacterial community in the gut through antibiotic use increased the susceptibility to pneumonia [4, 5] and supported a T-helper cell type 2 (Th2) response associated with allergic airway disease [6]. Similarly, germ-free mice show a decreased immunological tolerance and enhanced susceptibility for the development of allergic airway disease [7, 8].

With advanced age, the functioning of the immune system declines [9] and, as a result, infections and chronic inflammatory disease in the elderly are often associated with increased mortality [10, 11]. In a recent study we investigated age-related changes of allergic airway disease in a murine house dust mite (HDM) allergen model [12] and found a greater pulmonary allergic inflammation with a mixed Th2/Th17 response in old compared to young mice. Since the gut microbiota was shown to modulate allergic airway disease as well as a Th17 response (reviewed in [1]), it seems likely that intestinal bacteria impact age-related allergic responses. Recent studies have shown that the gut microbiome changes with age [13, 14] and a close relationship between healthy ageing, diet, and the composition of the gut microbiome was found [13]. Thus, age-related allergic inflammatory responses might be directly influenced by age-dependent alterations of the gut microbiota. However, investigations on associations between the microbial community, host age, and host immune senescence and (systemic) inflammation are in their infancy, but of critical importance in order to prevent/treat disease in the elderly.

In the current study we investigated the fecal microbiome of our murine model of allergic airway disease in both young and old mice based on* 16S rRNA* gene analysis. We were specifically interested in exploring if differences in the microbial community structure between young and old mice influence the magnitude and characteristics of allergic airway disease and whether, vice versa, the disease influences the community in the gut.

## 2. Materials and Methods

### 2.1. Murine Asthma Model

Male BALB/c mice were obtained from Harlan (Haslett, MI). Young mice were purchased at an age of 6–8 weeks and acclimated for at least one week before proceeding with the allergen protocol. Old mice were purchased at an age of 9-10 months (retired breeders) and aged for another five months at Michigan State University (MSU) animal housing facilities until experimental use. All animal procedures and experimental protocols were approved by the MSU Institutional Animal Care and Use Committee (IACUC).

HDM (allergen) was obtained from* Dermatophagoides pteronyssinus* whole body extract (Greer, Lenoir, NC; Lot # 165197). The animals were intranasally sensitized on days 1, 4, and 7 with 0 *μ*g (vehicle control) or 10 *μ*g HDM protein in a total volume of 30 *μ*L of saline. The mice were anesthetized with 4% isoflurane in O_2_ prior to each intranasal instillation. On day 21, HDM-sensitized mice were challenged intranasally with 20 *μ*g of HDM protein. All animals were sacrificed 48 h later on day 23. Animal sacrifice and necropsy was performed as described previously [12]. Each experimental group consisted of 6-7 mice.

### 2.2. Lung Histology

The left lung lobe was inflation-fixed with 4% paraformaldehyde, cut in random oriented serial sections, and embedded in paraffin. Paraffin sections were then stained with hematoxylin and eosin (H&E) and immunohistochemistry for major basic protein was performed as described previously [15].

### 2.3. Serum Analysis

Cytokines in the serum, namely, interleukin- (IL-) 1*β*, IL-5, IL-6, IL-10, IL-13, IL-17A, tumor necrosis factor alpha (TNF*α*), and interferon gamma (IFN*γ*), were measured with the enhanced sensitivity FLEX SET (200 fg to 200 pg/mL) Cytometric Bead Array (BD Biosciences, San Jose, CA). Cytokine analysis was performed using a FACSCalibur flow cytometer (BD Biosciences) and according to the FLEX SET manual. Serum cytokine data were reported as group means ± standard error of the mean (SEM). Grubbs outlier test was performed and significant outliers were excluded. Differences among groups were analyzed by a two-way ANOVA followed by a pairwise comparison with Student-Newman-Keuls test. Significance was assigned to *p* values ≤ 0.05. All analyses were conducted using SigmaPlot software (SYSTAT Software Inc., San Jose, CA).

### 2.4. Microbiome Analysis

Fecal pellets were excised from the colon at necropsy, snap frozen in liquid nitrogen, and stored at −80°C. DNA extraction was performed using the PowerSoil kit (MoBio, Jefferson City, MO, USA) according to the manufacturer. Primers, barcoding strategy, and amplification were done according to Kozich et al. [16]. Assembly of reads, chimera check, and read classification was described previously [17]. Ordination analysis (nonmetric multidimensional scaling (NMDS)) was performed in R (package:* vegan*) on Hellinger transformed data of relative abundances where reads were binned at the genus level. Samples were subsampled to 63730 reads before analysis.

## 3. Results

### 3.1. Old Mice Show Greater Allergic Airway Inflammation

**Figure 1:**
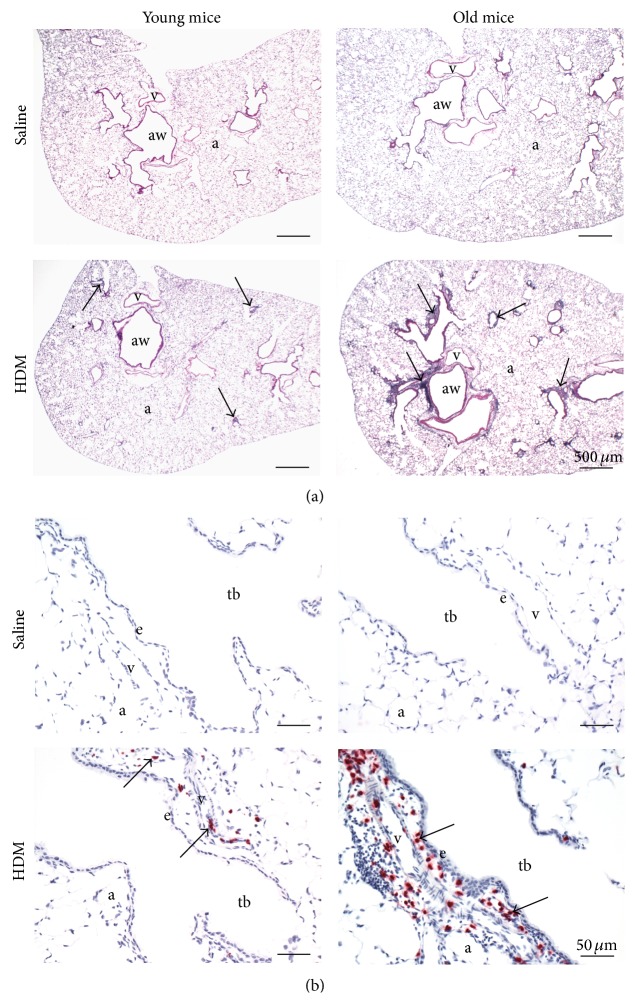
Pulmonary histopathology. (a) Representative light photomicrographs of lung tissue sections, stained with hematoxylin and eosin, showing a peribronchiolar and perivascular mixed inflammatory cell infiltration in HDM mice (arrows). aw: airways; a: airspace; v: blood vessel; scale bar: 500 *μ*m. (b) Accumulation of major basic protein positive eosinophils (arrow) in the perivascular and peribronchiolar region of young and old HDM mice. tb: terminal bronchioli; a: alveoli; v: blood vessel; scale bar: 50 *μ*m.

In both age groups of mice, intranasal sensitization and challenge with HDM (HDM mice) caused a peribronchiolar and a perivascular influx of inflammatory cells that was principally located in the proximal aspects of the lung lobe (Figure 1(a)). This HDM-induced mixed inflammatory cell influx was most prominent in the interstitial spaces surrounding pulmonary vessels (airway associated arteries/arterioles and veins/venules embedded in alveolar parenchyma) and large diameter preterminal bronchioles, with only occasional extension to the interstitium surrounding the small and more distal terminal bronchioles. Inflammatory cells were mainly mononuclear cells (lymphocytes and plasma cells) with lesser numbers of granulocytes. The latter group was composed of more major basic protein positive-eosinophils with lobulated nuclei than major basic protein-negative neutrophils with highly segmented nuclei. Neutrophils, however, were more conspicuous in the old compared to the young HDM mice. Light photomicrographs in Figure 1(b) illustrate the differences in the peribronchiolar accumulation of eosinophils among the experimental groups of mice.

### 3.2. Allergic Mice Have Elevated Serum IL-17A

We analyzed serum concentrations of IL-1*β*, IL-5, IL-6, IL-10, IL-13, IL-17A, TNF*α*, and IFN*γ*. Serum levels of IL-5 and IL-10 were below the assay detection limit (200 fg/mL). No significant differences were found with age or HDM exposure, except for IL-17A, which was induced in young HDM and old HDM mice. Systemic, HDM-specific IgG1 expression, as a marker of allergic response, was analyzed previously and showed a significant induction of IgG1 in HDM mice [12].

### 3.3. The Microbial Community in the Gut Changes with Age and HDM Exposure

Bacterial community compositions on the family level for individual samples are shown in Figure 3. All samples are dominated by few bacterial taxa, namely, the Porphyromonadaceae, Prevotellaceae, and Lachnospiraceae. A decrease of the Bacteroidetes to Firmicutes ratio was observed in old compared to young mice as well as for HDM mice compared to their corresponding controls (saline mice). This was mainly due to an increase in Lachnospiraceae (Firmicutes) at the expense of Porphyromonadaceae and Prevotellaceae (both Bacteroidetes). Ordination analysis clustered each experimental group separately (Figure 4) and both age and treatment were revealed as significant differentiation factors (*p* < 0.01 age, *p* < 0.05 treatment) (Figure 4). It has to be noted that the old mice in this study were all individually housed, whereas the young mice were housed in a group of three. However, no obvious cage effects were apparent, as the variation in community structures within and between cages in young animals was similar.

Detailed analysis on most abundant genera is displayed in Figure 5. Overall, a similar pattern was observed for old mice (compared to young animals) and HDM mice (compared to saline mice), where in particular* Barnesiella* sp.,* Prevotella* sp., and unclassified Porphyromonadaceae declined, whereas abundances of Lachnospiraceae* incertae sedis*, unclassified Clostridiales, and* Clostridium* XIVa and IV increased with age and HDM challenge. However, each comparison exhibited unique signatures; for example,* Odoribacter* sp. specifically decreased in young mice, while* Lactobacillus* sp. and* Desulfovibrio* increased with HDM treatment only in old mice. Several taxa showed considerable relative differences between groups, but only account for a small absolute abundances (Figures 5(a) and 5(b)), such as* Desulfovibrio* that increased by >150% in old HDM compared to saline mice, but represented an absolute difference of <0.2% (Figure 5(c)).

## 4. Discussion

Our study suggests that changes of the gut microbiome with age can have an impact on the development and manifestation of allergic airway disease. Furthermore, the results indicate that a locally induced pulmonary allergic response is affecting the composition of the intestinal microbiome suggesting that gut-lung interactions are bidirectional. These findings emphasize the importance of the gut-lung axis and strengthen its consideration for the development of treatment strategies for allergic airway disease.

A main known route of gut-lung interaction is mediated by regulatory T-cells (T-regs) as intestinally primed T-regs migrate to other mucosal tissues such as the bronchial epithelium, where they can modify the inflammatory response [18, 19]. T-regs have an immune-regulatory, anti-inflammatory function and are primed by microbe-derived products such as SCFAs, where especially propionate has a high T-reg priming potential [1, 3]. Thus, the decline in propionate-producing* Barnesiella* sp. and* Prevotella* sp. in old animals might be a plausible cause for the observed increased allergic inflammation in old HDM mice. However, investigations of T-cell populations in the spleen of these animals did not suggest a global change in T-reg numbers between young and old mice [12]. Moreover, we observed increasing abundances of taxa in old and allergic mice that were previously shown to correlate with anti-inflammatory response in the human gut, namely, Lachnospiraceae, Ruminococcaceae, and Rikenellaceae [20]. Thus, there is no indication that the enhanced allergic response in old mice is mediated by an age-dependent decrease in anti-inflammatory components of the gut microbiome. However, more functionally focused investigations on the gut microbiota are needed in order to draw definite conclusions on this topic.

**Figure 2:**
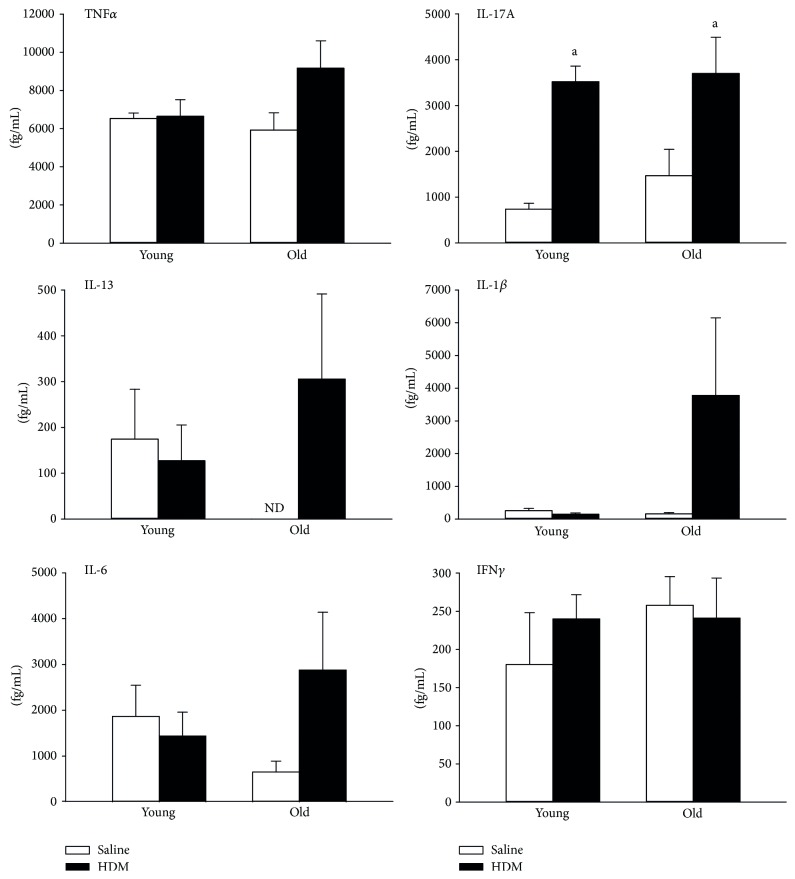
Expression of serum cytokines. Different serum cytokine concentrations were analyzed: TNF*α*, IFN*γ*, IL-1*β*, IL-6, IL-13, and IL-17A. a: significant changes (*p* < 0.05, *n* = 6-7) when compared to saline mice of the same age group; no significant changes were detected between young and old mice of the same exposure group.

**Figure 3:**
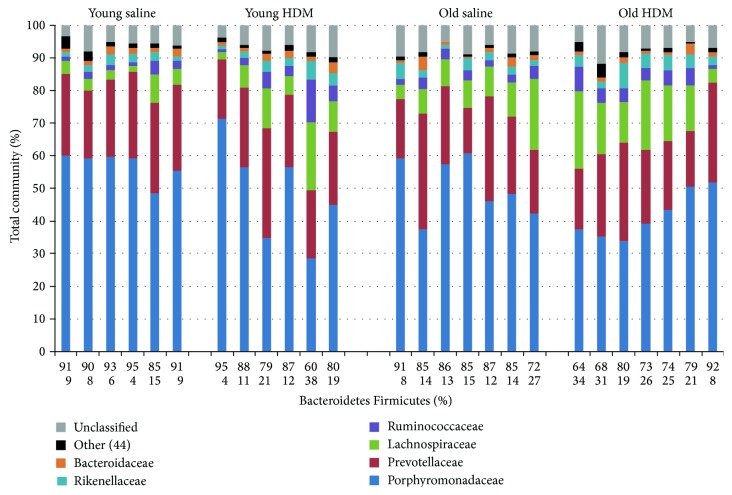
Fecal bacterial community structure at family level. Relative abundances of individual families as well as overall percentage of Bacteroidetes and Firmicutes are shown.

**Figure 4:**
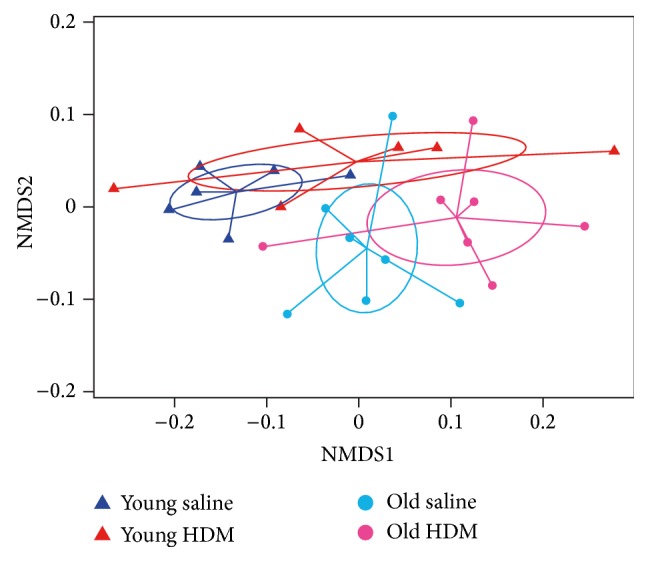
Nonmetric multidimensional scaling (NMDS) of bacterial communities. Saline mice (young: dark blue and old: light blue) and HDM mice (young: red and old: pink) are shown. Both age (*p* < 0.01) and HDM exposure (*p* < 0.05) significantly influenced community structure based on permutational ANOVA analysis. Ellipses represent the 95% confidence interval on standard errors of means.

Th17 cells provide another route of gut-lung interaction in allergic airway disease. Proinflammatory Th17 cells and immunotolerogenic T-regs develop from the same T-cell lineage dependent on the microbial and inflammatory milieu. Th17 response was shown to be involved in more severe forms of asthma [21] and to abrogate oral tolerance [22]. In this study we found IL-17A to be enhanced in the serum of both young and old HDM mice. Interestingly, only old HDM mice showed increased IL-17A levels in the BALF and enhanced Th17 cell numbers in the spleen [12]. Thus, the source for the observed increase of serum IL-17A after HDM exposure in young mice shown in Figure 2 is unknown. The observed IL-17 response to HDM might also source from a distinct body site not investigated here, such as the ileum where segmented filamentous bacteria (SFB) are known to promote Th17 differentiation with subsequent IL-17A production [23, 24]. SFB are not detectable in fecal pellets (also not detected here) and further investigations including ileal bacterial communities are needed in order to reveal their role in our HDM-allergy model.

Most studies in this field have solely focused on bacterial taxonomic markers (as done in this study) without addressing microbial functionality or more detailed investigations on host-microbiota interactions. Consequently, most of our knowledge on the gut microbiome and its effect on the human host is based on correlative investigations that are often governed by multiple factors that hamper the discovery of age-specific alterations in the gut microbiota and its impact on allergic airway disease. Furthermore, there is no clearly defined “microbiome of the elderly” in human, but rather a combination of ageing and life style that shapes the gut microbiome with age [13]. Animal studies can circumvent this multifactorial dilemma by excluding distinctive individual life style factors and our study did indeed reveal separate microbial communities in the two age groups. However, the mice were not raised their entire life-time in our facility (1 month or 6 months for young and old mice, resp.). Although, bacterial communities in the gut can adapt to external factors such as diet within only a few days [25], we cannot completely exclude the possibility of some structural varieties in the microbiome of young and old animals due to different housing times in our animal facility. The results presented here should, hence, be regarded as an initial reference. Eventually, fecal transplantation experiments are needed to prove that microbial communities predominating in older individuals can promote a proinflammatory phenotype.

**Figure 5:**
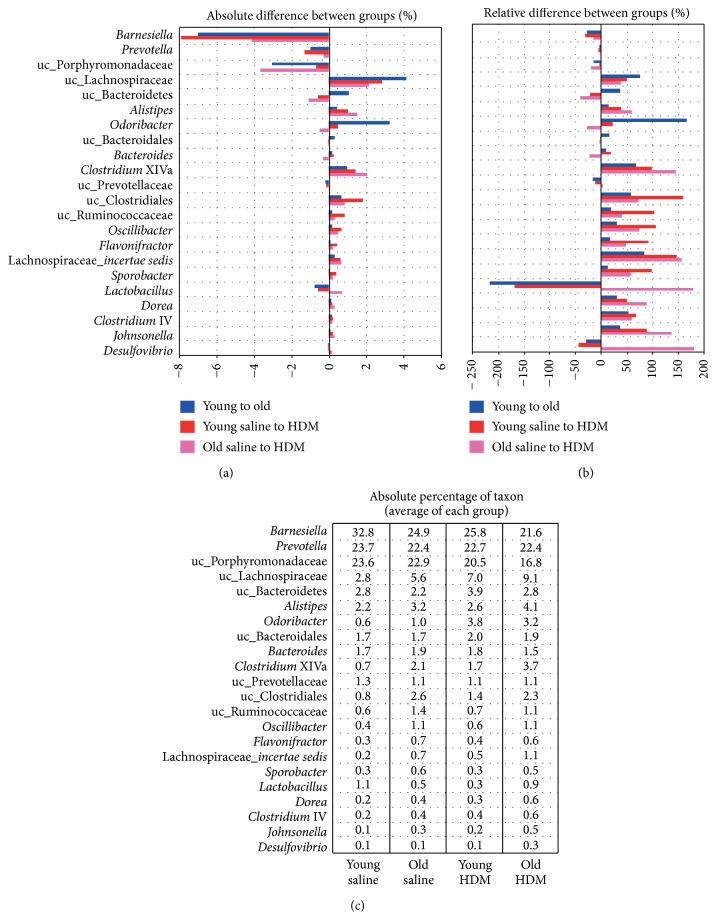
Details on community structure differences between groups. (a) shows the absolute difference of genera between groups (in %), whereas relative differences are given in figure (b). Relative abundances of taxa within groups are displayed in figure (c). Only taxa that comprised at least 1% of the total community in any sample are shown. uc: unclassified.

The HDM challenge in the lungs via intranasal administration had a profound impact on the gut microbiome structure in both young and old mice. This leads us to the hypothesis that the gut-lung axis is bidirectional, resembling a loop that can be stimulated from two sites. Thus, stimuli in the gut are transferred to the lung that provides a feedback to the gut, which again sends (altered) signals. Two different scenarios of lung-gut interactions are imaginable where stimuli from lung inflammation (i) alter the gut bacterial structure that further promotes an inflammatory phenotype or (ii) enhance specific gut microbes/production of metabolites that suppress proinflammatory signals (such as selection for specific SCFA-producers). In our model it is likely that signals from the inflamed lung direct alterations in the intestinal bacterial community structure that further support inflammation in the lung, since we found a similar shift in bacterial community structure after HDM exposure as with progressing age, which correlated with a stronger allergic response in old compared to young mice (Figure 5). Such continuous feedback stimulations might sustain inflammation in chronic diseases in the elderly. Interestingly, patients with chronic obstructive pulmonary disease (COPD) often show an increased prevalence of GI track diseases such as ulcerative colitis and Crohn's disease [26, 27] supporting our hypothesis of a proinflammatory cross talk between the lung and the gut. It has to be noted that small amounts of HDM could also be swallowed after intranasal administration and we cannot exclude that some HDM allergen reached the intestine influencing the local immunity of the gut. However, a previous study on oral administration of HDM in a mouse allergy model has shown that orally administered HDM induces levels of serum and fecal IgA and lowers levels of IgE [28], thus, rather promoting allergic tolerance than an allergic proinflammatory response.

With this study we provide first insights into the gut-lung interaction in an age-related allergy model. Our results indicate that the gut-lung axis is bidirectional and that both factors age and pulmonary allergy have an impact on the composition of the gut microbiota. It should serve as a stimulus for more mechanistically focused research and underlines the importance to consider multiple body sites for the treatment of disease.
